# Efficacy of Surgical Intervention in Treating Pathological Fractures of the Upper Extremity: A Retrospective Case Series

**DOI:** 10.7759/cureus.71273

**Published:** 2024-10-11

**Authors:** Kazuhiko Hashimoto, Shunji Nishimura, Tomohiko Ito, Ryosuke Kakinoki, Koji Goto

**Affiliations:** 1 Orthopedic Surgery, Kindai University Hospital, Osakasayama, JPN

**Keywords:** bone fractures, cancer, neoplasm metastasis, surgical procedures, upper extremity

## Abstract

Background: We conduct a retrospective analysis of patients with pathological fractures resulting from upper extremity malignancies, focusing on the evaluation of treatment strategies employed.

Materials and Methods: We retrospectively studied 10 patients with metastatic bone tumors of the upper extremities. The study variables included tumor site, primary pathology, duration from the first diagnosis of the primary lesion to the occurrence of the pathological fracture, use of bone-modifying drugs, surgical technique, adjuvant therapy, postoperative functional assessment, Katagiri’s score, American Society of Anesthesiologists physical status (ASA-PS), outcome, and correlations between the Eastern Cooperative Oncology Group Performance Status (ECOG-PS) and Musculoskeletal Tumor Society (MSTS) score.

Results: The sites involved were the humerus and radius in eight and two patients, respectively. Primary pathologies were liver cancer in three patients, lung cancer and renal cancer in two patients each, and one patient each with multiple myeloma, plasmacytoma, and Hodgkin’s lymphoma. Nine patients experienced pathological fractures, and one had an impending fracture. The median time from primary tumor diagnosis to fracture was 12.5 months. Bone-modifying drugs were administered in all cases. Surgical procedures included intramedullary nails in seven patients and plate fixation in two. Chemotherapy served as adjuvant therapy in nine cases. The mean MSTS score was 26.5, and Katagiri’s score averaged 6. The median ASA-PS stood at 2. Outcomes showed seven patients alive with disease and three dead from disease. A significant association between the ECOG-PS and MSTS score was not observed.

Conclusion: Pathological fractures caused by malignant bone tumors of the upper extremity should be treated proactively with surgery regardless of prognosis.

## Introduction

The most common cause of destructive bone lesions in adults is malignant bone disease, with the humerus being the second most frequent site for long-bone lesions [1,2]. In particular, metastatic fractures are a poor prognostic factor for increased mortality [3,4]. It has also been reported that the failure rate of surgical treatment for pathological fractures may be high [5]. While reoperation is disappointing for healthy patients, it is often disastrous for those who are seriously ill [5].

In contrast, it has also been reported that patients with pathological fractures greatly benefit from surgical treatment [6]. Although there have been several reports on the pathogenesis of humeral malignant bone tumors, little clinical information is available, and the details of this pathogenesis remain unknown [7,8]. Therefore, the treatment strategies for bone malignancies, including both metastases and primary lesions, as well as the use of bone-modifying drugs and the selection of surgical procedures, remain controversial [9]. Here, we report the outcomes of patients with malignant bone tumors of the upper extremities treated at our institution. Our aim was to provide a guide for treating pathological fractures of the upper extremity bones.

## Materials and methods

We retrospectively studied 10 patients with malignant bone tumors of the upper extremities who were treated at our hospital. Cases treated at the hospital from November 2018 to September 2023 were registered. The inclusion criteria consisted of patients with pathological or imminent fractures of the upper extremity who received treatment, including surgical intervention, at our hospital. Patients whose postoperative course could not be monitored were excluded from the study. The results are presented in Table 1.

**Table 1:** Series of patients. No.: number; M: male; F: female; IMN: intramedullary nail; ASA-PS: American Society of Anesthesiologists physical status; AWD: alive with disease; DOD: dead of disease.

No.	Age	Sex	Cite	Primary	Surgical treatment	Adjuvant therapy	Mirel’s score	Katagiri’s score	ASA-PS	Outcome	MSTS score	Follow-up periods (months)
1	75	M	Radius	Renal	-(RT)	-	9	6	1	AWD	27	4
2	83	F	Humerus	Liver	IMN	Chemotherapy (sorafenib)	9	7	2	DOD	27	20
3	56	M	Humerus	Lung	IMN	Chemotherapy (cisplatin and carboplatin)	9	6	2	AWD	26	1
4	70	M	Humerus	Renal	Double plating	Chemotherapy (sunitinib)	9	6	2	AWD	19	19
5	74	M	Humerus	Hodgkin’s lymphoma	IMN	Chemotherapy (Adriamycin, bleomycin, vinblastine, dacarbazine)	9	5	2	AWD	27	5
6	46	F	Humerus	Multiple myeloma	IMN	Chemotherapy (bortezomib)	9	6	2	AWD	28	6
7	77	F	Radius	Plasmacytoma	Plating	Chemotherapy (bortezomib)	9	3	1	AWD	25	2
8	74	M	Humerus	Liver	IMN	Chemotherapy (lenvatinib)	9	8	2	DOD	27	4
9	65	M	Humerus	Liver	IMN	Chemotherapy (lenvatinib)	9	4	2	AWD	25	3
10	73	M	Humerus	Lung	IMN	Chemotherapy (gefitinib)	9	4	1	DOD	25	16

Seven men and three women were enrolled in this study, with a median age of 73.5 (range: 17-83) years. This study is a descriptive case series and does not use statistical techniques. The study variables included the tumor site, primary pathology, duration from the first diagnosis of the primary lesion to the occurrence of the pathological fracture, use of bone-modifying drugs, surgical technique, adjuvant therapy, Musculoskeletal Tumor Society (MSTS) score [10], Katagiri’s score [11], and outcome. Surgical duration and intraoperative blood loss were analyzed. We also assessed the American Society of Anesthesiologists physical status (ASA-PS) before surgery [12]. The median duration of postoperative investigation was 4.5 (range: 1-20) months. The sites were the humerus and radius in eight and two patients, respectively. The primary pathology included liver cancer in three patients; lung cancer in two; one patient each with multiple myeloma, plasmacytoma, and Hodgkin’s lymphoma. Nine patients had pathological fractures, and one had an impending fracture. The median time from primary tumor diagnosis to pathological fracture injury was 12.5 (range: 0-190) months. Bone-modifying drugs were administered to five patients. Bisphosphonates were used in three patients, while denosumab and calcium preparations were used in one each. The mean Mirel’s score was 9 points for all patients. Surgical procedures included intramedullary nails (IMNs) and plate fixation in seven and two patients, respectively. The only other treatment was radiotherapy in one patient. Chemotherapy was used as adjuvant therapy in nine cases. 

Statistical analyses

The Eastern Cooperative Oncology Group Performance Status (ECOG-PS) and MSTS score were plotted, and a correlation diagram was drawn. The coefficient of determination (R^2^) was calculated by drawing an approximation line to assess the correlation between correlations. The strength of the correlation was determined according to Pearson's correlation coefficient (R) as follows: very strong, 1.0≥|R|≥0.7; strong, 0.7≥|R|≥0.5; moderate, 0.5≥|R|≥0.4; medium, 0.4≥|R|≥0.3; weak, 0.3≥|R|≥0.2; and no correlation, 0.2≥|R|≥0.0.

## Results

The study included 10 patients, with ages ranging from 46 to 83 years. The cohort consisted of six males and four females. The primary cancers identified were renal cancer (three patients), liver cancer (four patients), lung cancer (three patients), multiple myeloma (one patient), plasmacytoma (one patient), and Hodgkin’s lymphoma (one patient). Various surgical interventions were performed, including IMN for six patients, double plating for one patient, and plating for another. Two patients did not undergo surgical treatment. Adjuvant chemotherapy was administered to eight patients, while two patients did not receive any adjuvant therapy. Mirel's score was consistently high across all patients, indicating a significant risk of fracture. Katagiri’s scores varied, with most patients scoring between 5 and 9. The ASA-PS classification indicated that most patients were classified as ASA 1 or 2. Outcomes varied, with four patients experiencing "alive with disease" (AWD) and three "dead of disease" (DOD). The MSTS scores ranged from 19 to 28, reflecting varying degrees of functional outcomes post-treatment. Follow-up durations ranged from 1 to 20 months, providing insights into the short-term effectiveness of the treatments administered.

**Figure 1:**
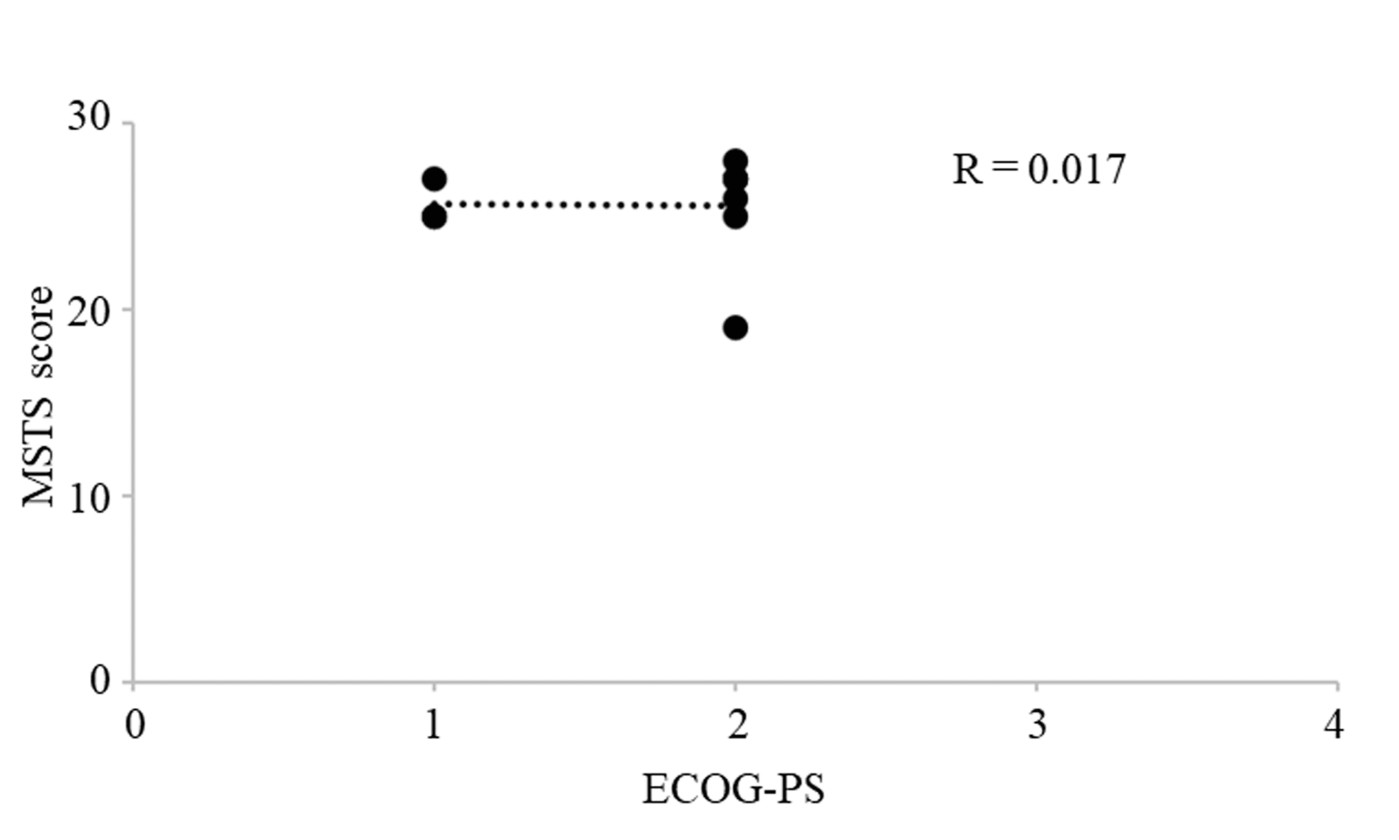
Graphs showing no significant correlation between the MSTS score and ECOG-PS (R=0.017). ECOG-PS: Eastern Cooperative Oncology Group Performance Status; MSTS: Musculoskeletal Tumor Society.

The mean MSTS score for postoperative function was 26.5 (range: 19-28). The mean Katagiri’s score was 6 (range: 3-8). The median ASA-PS was 2 (range: 1-2). The outcomes included seven patients AWD and three patients DOD. No significant correlation was observed between ECOG-PS and the MSTS scores (R=0.017, Figure 1).

The surgical time was 98±23.1 minutes (56-126 minutes, mean±SD). The blood loss was 20±28.6 mL (20-111 mL, mean±SD). No surgical complications or bone fusions were observed.

**Figure 2:**
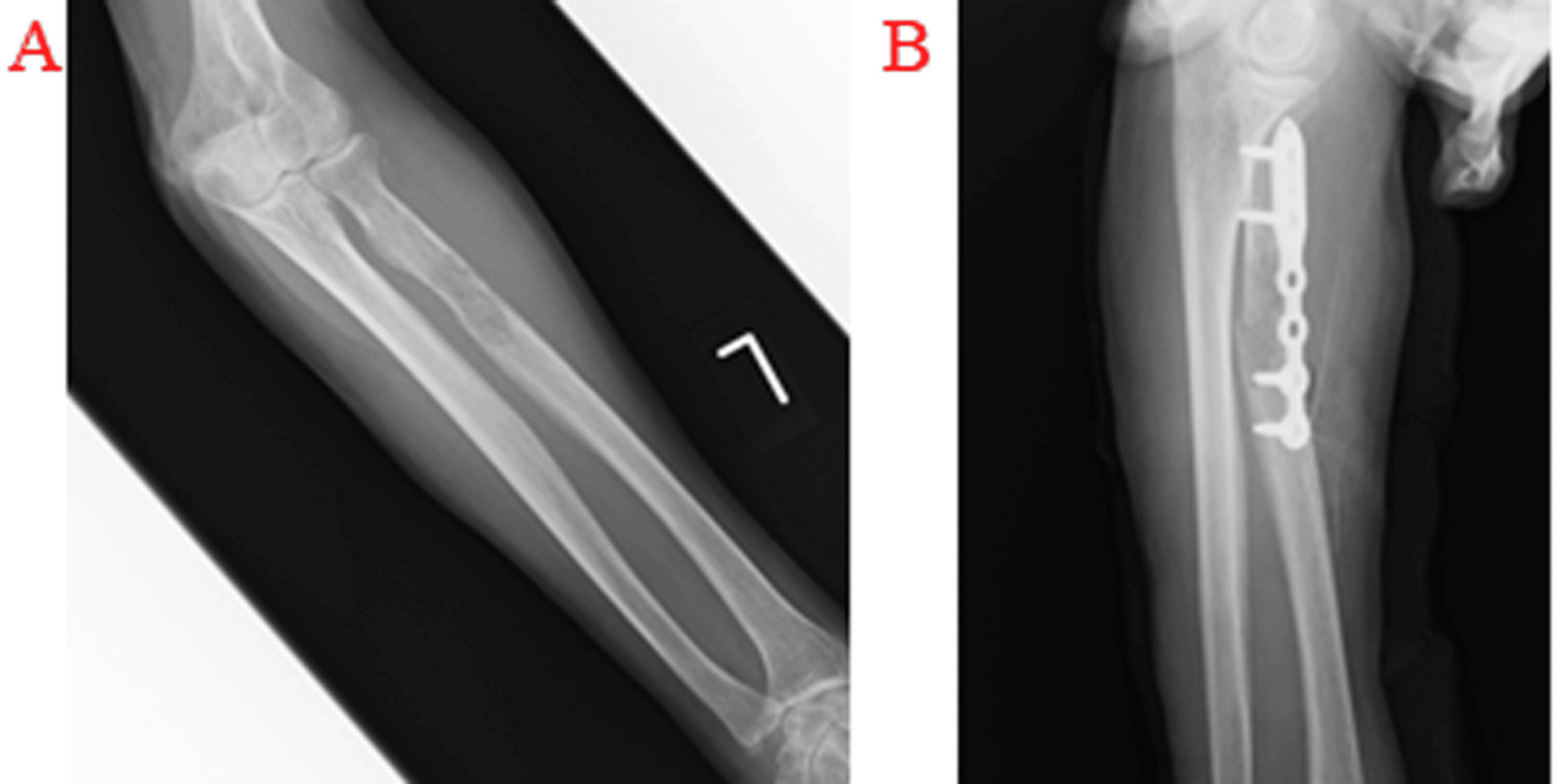
Radiographic findings of a pathological fracture of the proximal left radius. (A) The primary lesion was plasmacytoma with osteolysis. (B) Radiographic findings after fixation of the lesion with a plate.

Three representative cases are presented. The first case was a 77-year-old woman who complained of swelling and pain in her left forearm for two to three weeks. She came to our department because she could not move her left arm due to pain after falling on her hand. Radiography showed a fracture of the proximal radius and osteolysis (Figure 2A). An incisional biopsy and plate fixation were performed (Figure 2B). The pathology was plasmacytoma. After four weeks of postoperative immobilization, range-of-motion training of the elbow was performed. The affected limb recovered function at eight weeks postoperatively.

**Figure 3:**
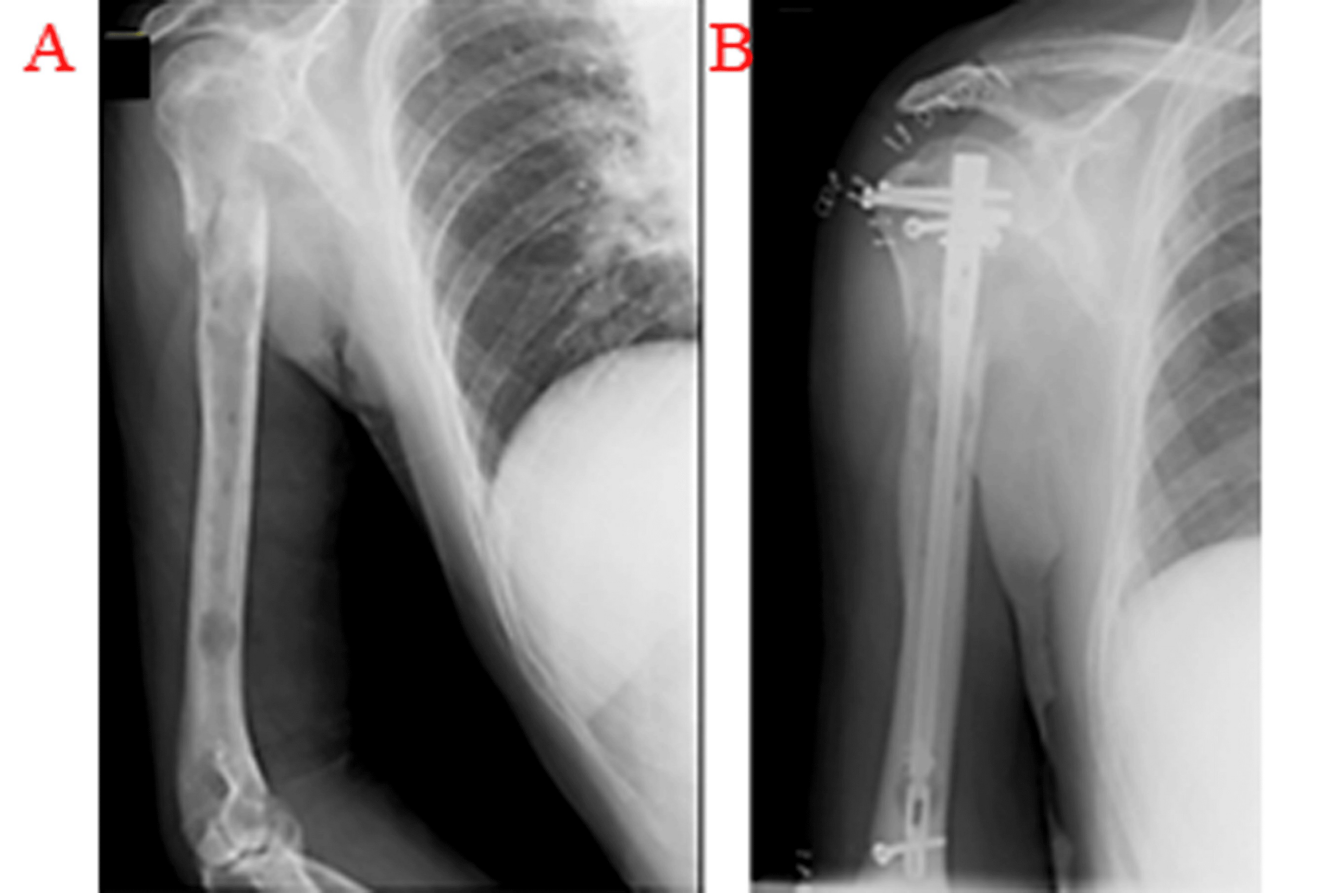
Radiographic findings of a pathological fracture of the right humerus. (A) The primary lesion was lung cancer with osteolysis. (B) Radiographic findings after treatment with IMN. IMN: intramedullary nail.

The second case involved a 73-year-old man. He had been undergoing chemotherapy for lung cancer for the past one year and had noticed pain in his right shoulder and elbow for three months. He was unable to move his right upper extremity after holding an object, so he visited our department. Radiography indicated a pathological fracture and bone osteolysis of the right humerus (Figure 3A). Fixation with an IMN was performed (Figure 3B). Four weeks later, the range of motion of the right shoulder joint had improved, and the elbow joint could be used. The patient died 16 months after surgery.

**Figure 4:**
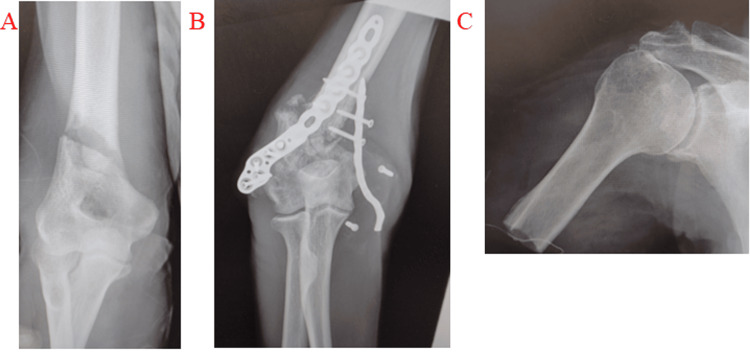
A 70-year-old man with kidney cancer. The patient sustained a pathological fracture of the right humeral condyle (A). The fracture was initially fixed with a double plate. However, the tumor continued to grow, causing the fracture (B). Consequently, the plate was removed, and amputation of the arm at the proximal humerus was performed (C).

The third case involves a 70-year-old male patient with kidney cancer who sustained a pathological fracture of the right humeral condyle (Figure 4A). Initially, double plate fixation was performed (Figure 4B). However, six months later, the tumor had grown, causing the plate to dislocate. Ultimately, amputation of the arm at the proximal humerus was necessary (Figure 4C).

## Discussion

Numerous uncertainties remain concerning pathological fractures of the upper extremities resulting from cancer metastasis and primary lesions. This is due to the limited literature available on the subject. This study reviewed the outcomes of patients with upper extremity malignant bone tumors treated at our institution.

Bones are one of the most common sites of metastasis from advanced solid tumors, and metastatic tumors in bones occur in 65%-80% of patients with advanced prostate or breast cancer, 40%-50% of patients with lung cancer, and <10% of patients with digestive cancers [13-15]. Other types of cancer that are prone to bone metastasis include esophageal cancer, malignant lymphoma, and renal cancer [16-18]. The incidence of skeletal metastasis in liver cancer is approximately 25% [19]. The median time from initial cancer diagnosis to bone metastasis is 18.9 months [19]. Liver cancer was relatively common in this study.

This study assessed the risk of bone fracture. Bone fractures were treated as early as possible; however, the patients developed pathological fractures at a relatively early stage. Previously, pathological bone fractures caused by metastatic bone tumors were treated conservatively in patients with end-stage cancer [20,21]. Harrington [22] listed the following criteria for surgery: life expectancy of at least two months, ability to tolerate surgery, recovery from surgical function, easy access to nursing care, and ability to stabilize and support the fracture with a metal fixture. In addition, Mirel’s score recommends prophylactic IMN fixation at a score of 8 or higher; however, some studies recommend surgery at a lower score, which is controversial [23,24].

In this study, regardless of the prognostic duration, fixation with implants was performed if the patient's general condition was amenable to surgery. One patient who was treated conservatively with radiotherapy was considered ineligible for surgery because the lesion was large and plate fixation did not provide joint stability. All patients were eligible for surgery according to Mirel’s score. However, at 9 points, the patients had already developed pathological fractures. Mirel’s score is only used as a reference, and indications should be considered in the current era of medical advances. If improvement in activities of daily living through functional recovery is desired, surgery should be performed regardless of prognosis as long as the patient’s general condition permits.

The surgical treatment of pathological fractures of the upper extremities includes IMNs and endoprostheses, both of which have good functional outcomes [25,26]. A comparison between IMNs and endoprostheses revealed that endoprostheses had a better long-term functional prognosis [26]. For patients in which neither surgery nor radiotherapy is possible, the IlluminOss® System based on photodynamic bone stabilization is a recent option among the minimally invasive surgical techniques available for treating bone metastases [27]. Compared to our outcomes for pathological fractures of the lower extremity, surgical treatment of pathological fractures of the upper extremity is relatively less invasive [28]. This is evidenced by shorter surgical times and reduced blood loss.

The functional results of this study, in which all but one patient underwent surgery, were also favorable. The results are generally favorable if the treatment is tailored to the patient’s needs. It may also be necessary to perform surgery more aggressively.

Bone metastases differ depending on the cancer type [28]. For example, patients with stage IV breast cancer have different clinicopathological characteristics and survival outcomes depending on the site of metastasis [29]; patients with bone metastases had the best prognosis [29], whereas those with renal cancer had a long-term prognosis [2]. Katagiri’s score reports a one-year survival rate greater than 80% for 0-3 points, 30%-80% for 4-6 points, and ≤10% for 7-10 points [11]. To reduce the risk of reoperation, the importance of identifying patients who are expected to survive long-term has been reported [5]. It has also been reported that patients with a good prognosis should be considered for extensive resection and reconstruction, as applied to primary malignant bone tumors [5]. The median Katagiri's score reported in this study was 6 points, and the one-year survival rate ranged from 30% to 80%. We recommend aggressive surgery even if the patient has less than six weeks to live, as long as the patient can tolerate the anesthesia. There was one case in this study in which the patient died four weeks after surgery. In addition, postoperative MSTS scores did not correlate with preoperative ECOG-PS, suggesting that a poor preoperative ECOG-PS did not affect MSTS scores. This provides evidence to recommend surgical treatment regardless of preoperative status. Radical treatment should be used to re-establish activities of daily living as much as possible. As previously reported, the overall one-year patient survival rate ranges from 42% to 75% [30]. In this study, the survival rate is relatively favorable, suggesting that treatment decisions should be made with the expectation of long-term survival.

## Conclusions

The conclusion of this study on the efficacy of surgical intervention in upper extremity pathological fractures suggests that these fractures, resulting from malignant bone tumors, should be treated with surgery, regardless of the patient's prognosis. It indicates that surgical treatment can significantly improve the quality of life and functional outcomes for patients, even those with a limited life expectancy. The findings support the notion that surgical intervention is beneficial for maintaining daily living activities in patients with terminal conditions. This study highlights the importance of tailoring treatment to individual patient needs and suggests that surgery should be considered as a viable option for enhancing patient comfort and functionality during their remaining life.
